# Endoscopic removal of a large gastric trichobezoar using a snare modified from a lithotripter to facilitate argon plasma coagulation

**DOI:** 10.1055/a-2562-4179

**Published:** 2025-04-03

**Authors:** Zhenghua Wang, Bing Bai, Xinru Zhang, Ran Li, Yayong Chen, Yahui Chen, Bin Li

**Affiliations:** 1 34708 Gastroenterology, Shandong Provincial Hospital Affiliated to Shandong First Medical University, Jinan, China; 2 Digestive Diseases Hospital of Shandong First Medical University, Jining, China

Trichobezoars, rare gastric bezoars made of hair, are most common in young girls with psychiatric disorders. They usually accumulate in the stomach and may extend into the small intestine, known as Rapunzel syndrome 1 . Patients often present with obstruction, bleeding, or perforation. Larger trichobezoars typically require surgery due to the challenges of endoscopic treatment 2 3 4 . Here, we report a successful endoscopic retrieval of a large gastric trichobezoar using a snare made from the core of a lithotripter to assist in argon plasma coagulation ( Video 1 ).

Endoscopic removal of a large gastric trichobezoar using a snare created with a modified lithotripter to facilitate argon plasma coagulation in a 16-year-old girl.Video 1

A 16-year-old girl with autism presented with upper gastrointestinal obstruction. Endoscopy showed a large gastric trichobezoar mixed with string and plastic ( Fig. 1 ). The trichobezoar extended from the gastric fundus to the pylorus, partially into the descending part of the duodenum ( Fig. 2 ), making it too large for a polypectomy snare or lithotripter to encircle.

**Fig. 1:**
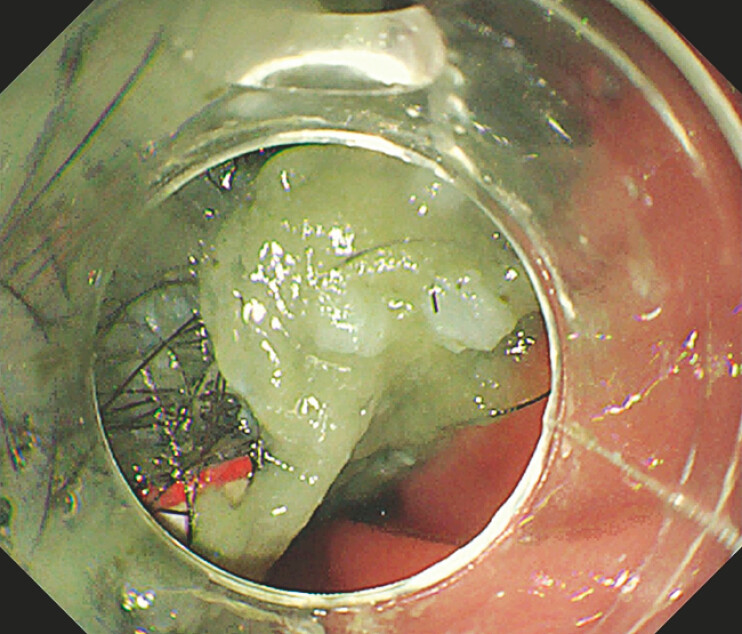
Endoscopic image of a large gastric trichobezoar mixed with string and plastic in a 16-year-old girl.

**Fig. 2:**
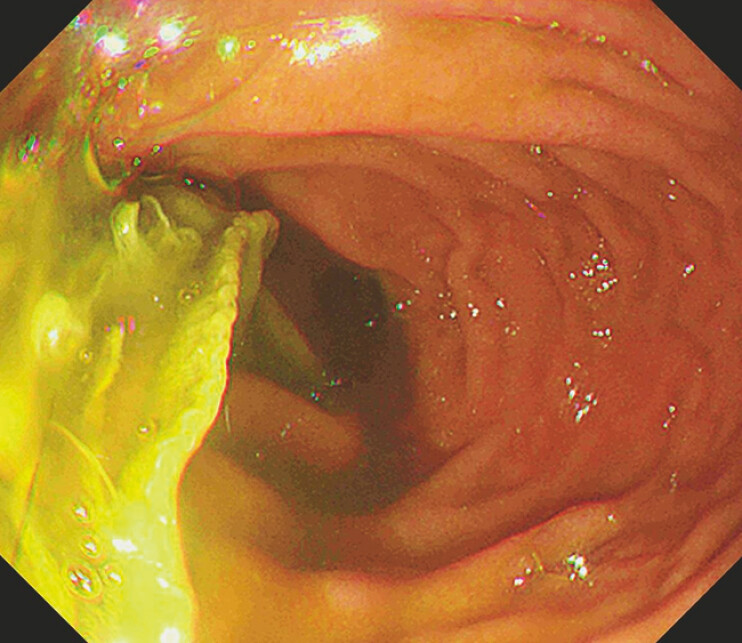
The trichobezoar is seen extending from the gastric fundus to the pylorus, with partial protrusion into the descending part of the duodenum.

To address this, we modified the lithotripter by cutting the core wire and forming loops at both ends to create a snare wire ( Fig. 3 ). First, one loop was secured to the anterior gastric wall near the location of the trichobezoar using a titanium clip ( Fig. 4 **a** ). An overtube was placed, and a polypectomy snare was positioned over the transparent cap before advancing the endoscope. The other loop was held with foreign body forceps and passed from the posterior wall of the stomach along the greater curvature, encircling the trichobezoar, until it converged with the loop fixed to the anterior wall ( Fig. 4 **b, c** ). The snare was released to encircle the wire loop, and both loops were grasped with forceps ( Fig. 4 **d, e** ). The forceps and snare were tightened, guiding the trichobezoar to the lower esophagus, and the endoscope was withdrawn ( Fig. 4 **f** ). The other endoscope was inserted and argon plasma coagulation was applied along the snare wire to fragment the trichobezoar. By repeating the aforementioned steps, the trichobezoar was completely removed ( Fig. 5 ).

**Fig. 3:**
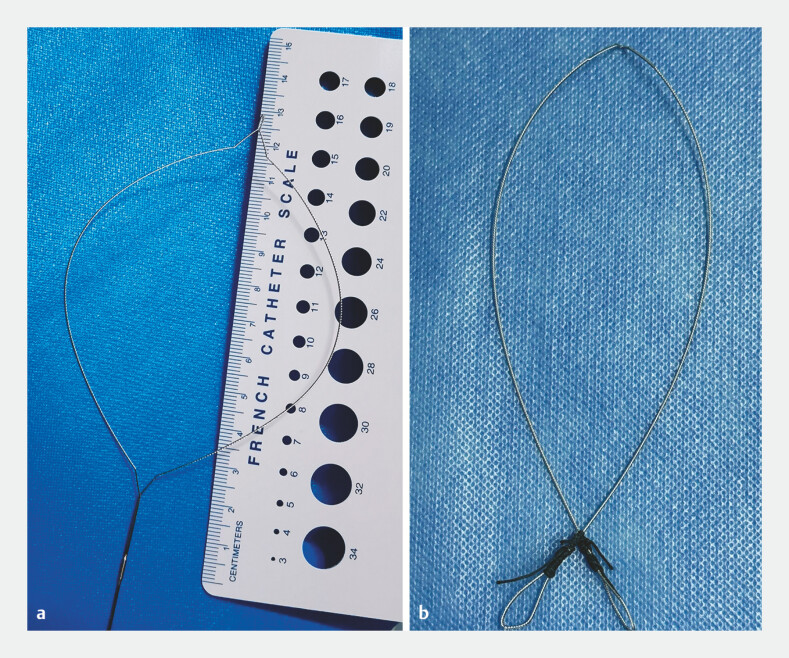
**a** Core of the lithotripter. **b** The lithotripter was modified by cutting the core wire and forming loops at both ends to create a snare wire.

**Fig. 4:**
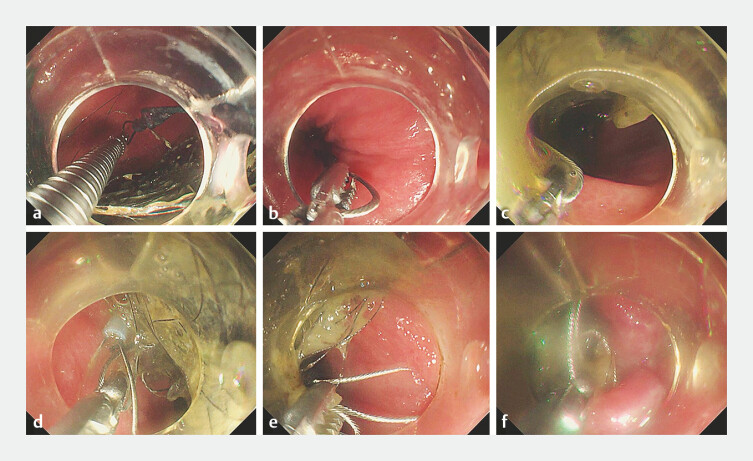
Endoscopic retrieval of the gastric trichobezoar. **a** One loop was secured to the anterior gastric wall near the trichobezoar using a titanium clip. **b** The second loop was grasped with foreign body forceps. **c** The loop was maneuvered around the base of the trichobezoar and joined with the loop fixed to the anterior wall. **d** The polypectomy snare was released over a transparent cap. **e** The two loops were grasped using foreign body forceps. **f** The forceps and snare were tightened, guiding the trichobezoar into the lower esophagus.

**Fig. 5:**
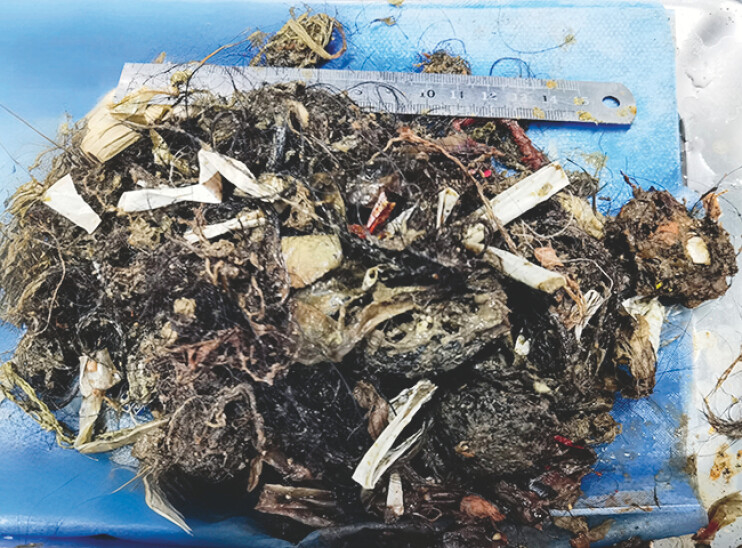
The trichobezoar was completely removed following fragmentation.

Endoscopy_UCTN_Code_TTT_1AO_2AL

## Notes

Endoscopy E-Videos https://eref.thieme.de/e-videos
*E-Videos* is an open access online section of the journal *Endoscopy* , reporting on interesting cases and new techniques in gastroenterological endoscopy. All papers include a high-quality video and are published with a Creative Commons CC-BY license. Endoscopy E-Videos qualify for HINARI discounts and waivers and eligibility is automatically checked during the submission process. We grant 100% waivers to articles whose corresponding authors are based in Group A countries and 50% waivers to those who are based in Group B countries as classified by Research4Life (see: https://www.research4life.org/access/eligibility/ ). This section has its own submission website at https://mc.manuscriptcentral.com/e-videos .
